# Bladder cancer cells secrete while normal bladder cells express but do not secrete AGR2

**DOI:** 10.18632/oncotarget.7400

**Published:** 2016-02-15

**Authors:** Melissa E. Ho, Sue-Ing Quek, Lawrence D. True, Roland Seiler, Achim Fleischmann, Lora Bagryanova, Sara R. Kim, David Chia, Lee Goodglick, Yoshiko Shimizu, Charles J. Rosser, Yuqian Gao, Alvin Y. Liu

**Affiliations:** ^1^ Department of Urology, Institute for Stem Cell and Regenerative Medicine, University of Washington, Seattle, WA, USA; ^2^ Department of Pathology, University of Washington, Seattle, WA, USA; ^3^ Department of Urology, University Hospital of Bern, Bern, Switzerland; ^4^ Institute of Pathology, University Hospital of Bern, Bern, Switzerland; ^5^ Department of Pathology and Laboratory Medicine, David Geffen School of Medicine, University of California, Los Angeles, CA, USA; ^6^ Jonsson Comprehensive Cancer Center, David Geffen School of Medicine, University of California, Los Angeles, CA, USA; ^7^ University of Hawaii Cancer Center, Honolulu, HI, USA; ^8^ Biological Sciences Division, Pacific Northwest National Laboratory, Richland, WA, USA; ^9^ Present address: University of California San Francisco Medical Center, San Francisco, CA, USA; ^10^ Present address: Singapore Polytechnic, Center for Biomedical & Life Sciences, Singapore

**Keywords:** secreted AGR2, bladder cancer, subcellular localization, urine biomarker

## Abstract

Anterior gradient 2 (AGR2) is a cancer-associated secreted protein found predominantly in adenocarcinomas. Given its ubiquity in solid tumors, cancer-secreted AGR2 could be a useful biomarker in urine or blood for early detection. However, normal organs express and might also secrete AGR2, which would impact its utility as a cancer biomarker. Uniform AGR2 expression is found in the normal bladder urothelium. Little AGR2 is secreted by the urothelial cells as no measurable amounts could be detected in urine. The urinary proteomes of healthy people contain no listing for AGR2. Likewise, the blood proteomes of healthy people also contain no significant peptide counts for AGR2 suggesting little urothelial secretion into capillaries of the lamina propria. Expression of AGR2 is lost in urothelial carcinoma, with only 25% of primary tumors observed to retain AGR2 expression in a cohort of lymph node-positive cases. AGR2 is secreted by the urothelial carcinoma cells as urinary AGR2 was measured in the voided urine of 25% of the cases analyzed in a cohort of cancer *vs*. non-cancer patients. The fraction of AGR2-positive urine samples was consistent with the fraction of urothelial carcinoma that stained positive for AGR2. Since cancer cells secrete AGR2 while normal cells do not, its measurement in body fluids could be used to indicate tumor presence. Furthermore, AGR2 has also been found on the cell surface of cancer cells. Taken together, secretion and cell surface localization of AGR2 are characteristic of cancer, while expression of AGR2 by itself is not.

## INTRODUCTION

Anterior gradient 2 (AGR2) is an adenocarcinoma antigen with elevated expression in many solid tumor types including prostate, breast, pancreatic, gastro-intestinal, lung. The protein is a protein disulfide isomerase (PDI) found in the endoplasmic reticulum (ER) where it functions as a molecular chaperone in protein folding [1]. The ER could have an important role in carcinogenesis and tumor biology [2]. A non-canonic ER retention motif (*C*-terminal KTEL) in AGR2 may be responsible for the diverse trafficking of this molecule [3]. In addition to the ER, AGR2 can be shunted to the nucleus, cell surface or extracellular space. Secreted AGR2 can function to trigger cellular differentiation in responding cells as documented in salamander limb regeneration [4]. AGR2 is secreted as a 19 kDa protein by prostate cancer cells measurable at pg/ml levels in the urine [5], and blood [6] of patients. AGR2 was identified as a biomarker candidate in prostate cancer by comparative transcriptomic analysis of sorted CD26^+^ cancer cells *vs*. CD26^+^ luminal cells [7]. In this approach, genes with elevated expression encoding secreted proteins in cancer are considered to be viable candidates; genes expressed by both cancer and normal cell types are not, and are thus ignored. A further consideration is that AGR2 expressed in the cancer of one organ could be expressed in the normal tissue of other organs, which would impact its utility as a biomarker. There would be very few cancer-specific biomarkers based on expression difference if examined systematically between cancer and all normal tissue. This is not unexpected since every gene in the genome serves a useful function in some cell types. AGR2 is secreted by pancreatic cancer cells [8], and localized on the cell surface of pancreatic cancer cells [9]. Whether normal AGR2-positive cells also secrete or express this protein on the cell surface is not known. Both normal prostate and pancreatic epithelial cells do not express AGR2 [7, 9].

In this study, urinary bladder expression of AGR2 was investigated. Cell-type transcriptomes previously indicated that CD9^+^ urothelial cells were positive for AGR2 expression [10]. The entire urothelium was immunostained positive for AGR2. However, AGR2 was not observed to be secreted by the urothelial cells as little of this protein was detected in urine [5, 6]. This finding was supported by database query of urine proteomes containing urinary proteins identified by large-scale mass spectrometry proteomics. Database query of blood proteomes similarly indicated little AGR2 in circulation as, for example, from secretion by urothelial cells into capillaries within the underlying lamina propria. AGR2 expression is lost in urothelial carcinoma, but a minority of bladder tumors were found to retain AGR2. The detection of AGR2 in the urine of a subset of bladder cancer patients indicates that AGR2 could be secreted by urothelial carcinoma cells. The take home message is that differential subcellular localization or protein address of AGR2 – cell interior *vs*. cell exterior – not expression, makes AGR2 a cancer biomarker. This may reflect the different functional roles played by AGR2 in normal cells *vs*. cancer cells. Such localization differences appear to be a property of PDI enzymes [11]. Cell surface expression could be inhibited by Brefeldin A [12]. In clinical utility, AGR2^+^ cancer cells can be targeted for eradication by agents such as antibodies or antibody drug conjugates, while AGR2^+^ normal cells would be spared.

## RESULTS

### Expression of AGR2 by urothelial cells

**Figure 1:**
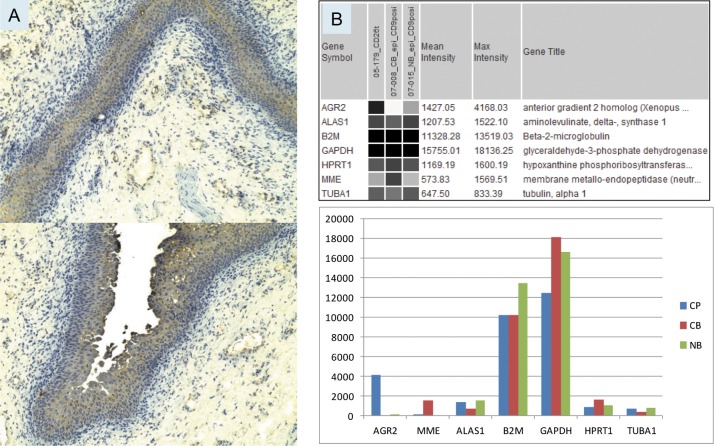
Bladder AGR2 expression **A.** The urothelial cells in specimen 03-043B1 are stained at moderate intensity (compared with that in prostate cancer cells) by anti-AGR2. Two different parts of the tissue specimen show uniform expression likely throughout the urothelium. Magnification is 100x. **B.** Transcriptome dataset query display compares the expression levels of AGR2 and reference genes in sorted populations of prostate cancer cells (05-179_CD26t), bladder cancer cells (07-008_CB_epi_CD9posi), and normal bladder cells (07-015_NB_epi_CD9posi). The same data is shown below in a histogram format – CP = prostate cancer; CB = bladder cancer; NB = normal bladder. Probeset intensity values are on the *y*-axis.

The normal bladder urothelium was uniformly immunostained for AGR2 at moderate intensity as shown in Figure 1A. Immunostaining of prostate cancer cells was comparatively more intense [13, 14]. This immunostaining confirmed the gene expression detected by DNA microarray analysis of sorted CD9^+^ urothelial cells. Both paraffinized sections stained with polyclonal anti-AGR2 and frozen sections stained with monoclonal anti-AGR2 showed that the two antibodies recognized the same cell types. Urothelial AGR2 expression was recorded in five benign tissue specimens. Urothelial AGR2 expression was 40 times lower than prostate cancer AGR2 expression as calculated from the array signal intensity values for AGR2 in CD9^+^ urothelial cells and sorted CD26^+^ prostate cancer cells (Figure 1B). A good correlation between array signal values and immunostaining intensity in our biomarker study was reported previously [15]. Values for the selected reference genes were all found to be equivalent. In addition, the observation of faint AGR2 staining of the stroma (in frozen sections) next to the prostate tumor glands could be used to indicate that AGR2 was secreted [14]. No such staining was seen in the stroma adjacent to the urothelium (Figure 1A) suggesting that AGR2 was not secreted by the urothelial cells.

### Bladder cancer expression of AGR2

**Figure 2:**
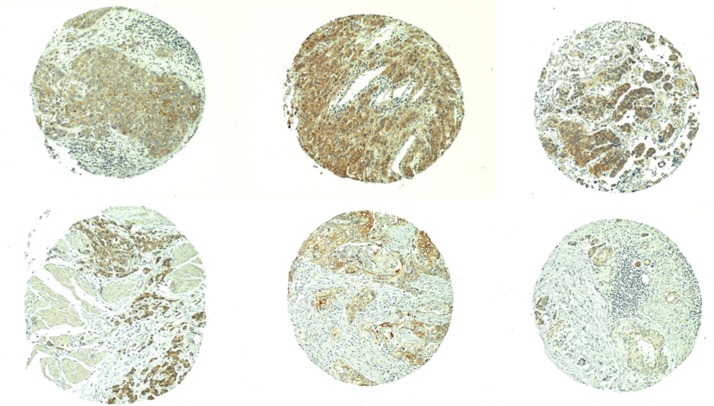
AGR2 in primary bladder cancer Shown are TMA examples of tumor immunostaining for AGR2. Positive cells are in brown.

AGR2 expression in bladder cancer was examined in a cohort of patients with lymph node involvement. In 129/152 evaluable spots of tissue taken from the centers of primary tumors, 24% showed AGR2 immunostaining, whereas in 124/152 evaluable spots taken from the tumor invasion fronts, 12% showed AGR2 immunostaining. Examples of AGR2 tumor immunostaining are shown in Figure 2. In many cases, the cancer staining appeared stronger than that of normal urothelial cells. Thus, in a majority of analyzed bladder cancer cases, malignant transformation was accompanied by a loss of urothelial AGR2. The microarray result of sorted CD9^+^ cancer cells from one bladder tumor specimen showed no AGR2 expression (Figure 1B), but showed CD10 expression (Figure 1B). CD10 was investigated because of its importance in lymph node spread of prostate cancer cells, and its diametrically opposite role to that of AGR2 in the local *vs*. distal spread of tumor [16]. Most AGR2^+^ prostate cancer cells were negative for CD10 [14]. Normal AGR2^+^ urothelial cells also showed no CD10 expression [10].

**Figure 3:**
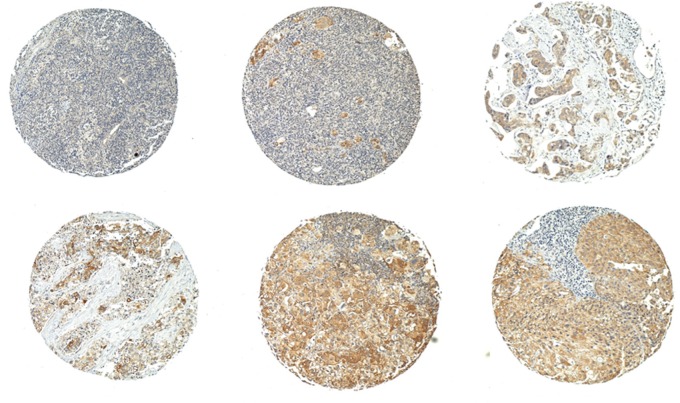
AGR2 in lymph node metastases Shown are TMA examples of lymph node immunostaining for AGR2. The amount of cancer cells is variable in the specimen cores taken for the tissue array.

In 125/152 and 121/152 evaluable spots of lymph node metastases on two TMA, 44% showed AGR2 staining, examples of which are shown in Figure 3. There were instances in which AGR2 staining was not detected in the primary tumor but was detected in the corresponding lymph nodes (e.g., cases B97-05, B00-08, B05-05, Supplementary Table 1). Many of the primary tumors showing AGR2 staining also showed AGR2-positive lymph nodes. McNemar's test with Yates' correction for continuity for this distribution of AGR2 positivity between primary tumors and lymph node metastases was χ^2^ = 9.24 (*P* = <0.01).

There was no correlation observed between patient survival and AGR2 expression: *P* = 0.475 for AGR2^+^ tumor center, *P* = 0.387 for AGR2^+^ invasion front in a univariate analysis; *P* = 0.39 and 0.73, respectively, in a multivariate analysis. In contrast, capsule perforation plus age, gender, and pT stage were significant predictors of survival in agreement with our previous study results [17]. When the patients were divided into >10 y survival groups (n = 10, 6.6%) and <1 y survival (n = 42, 27.8%), most of the long survival cases (in spite of their positive lymph node status) showed absent or low AGR2 staining in the primary tumor with the exception of case B94-01 (Supplementary Table 1). Although B94-01 was staged pT4 and pN2, there was no capsule perforation, which was the best indicator of survival. In the poor survival group, both AGR2^+^ and AGR2^−^ tumors were observed.

### Urinary AGR2

**Figure 4:**
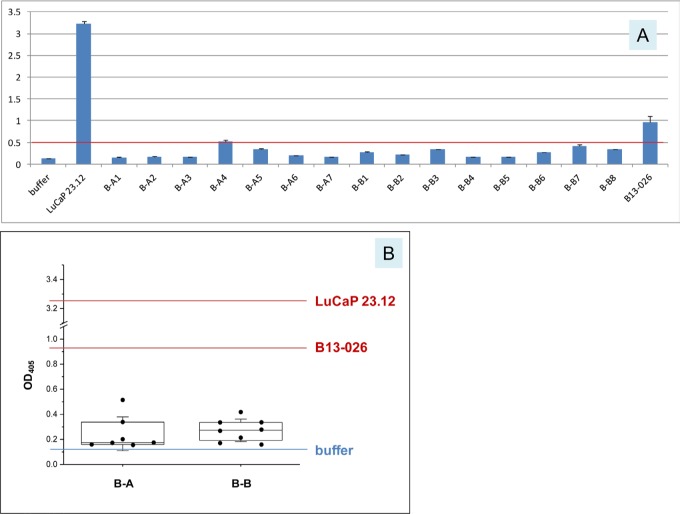
Urinary AGR2 levels in healthy women **A.** In the histogram, OD_405_ readings are on the *y*-axis for the samples listed on the *x*-axis. B-A1 to B-A7 are seven donations from female subject A (the prefix B is for “bladder” subject urine *vs*. P for “prostate” subject urine); B-B1 to B-B8 are eight donations from female subject B. The red line marks the highest level (B-A4) measured for these non-cancer urine. LuCaP 23.12 is the cell-free tissue digestion media of the prostate cancer xenograft. B13-026 is the urine from a bladder cancer patient. **B.** In the boxplot, the 25 and 75 percentile values and the medians are shown. The standard deviation (SD) values are shown using whiskers. For comparison, the values for LuCaP 23.12 and B13-026 are included.

Voided urine samples from two healthy female donors (B-A and B-B) collected on different days were assayed for AGR2. The levels of AGR2 observed in both urine samples were close to the buffer background (Figure 4). The positive control of collagenase digestion media of prostate cancer xenograft LuCaP 23.12 tumor contained a level of AGR2 at 25-fold higher than that of the buffer. High AGR2 expression in LuCaP 23.12 was previously shown by immunostaining and DNA array analysis [14]. Despite the entire urothelium being positive for its expression, little of the small 19 kDa AGR2 was released by the bladder into urine. No AGR2 was detectable by Western blotting of urine samples [13]. This conclusion was supported by urine proteome database queries. No match was found for AGR2 in the *UrinePA-PeptideAtlas* of 2,500 proteins identified by proteomics. AGR2 was not found in the core urinary proteome of healthy people. Queries of other recently published normal urine proteomes (e.g., ref. 18) also revealed no data entry for AGR2. For comparison, UPK3A (uroplakin) from bladder cells had 2 identifiers in 3 builds, and was observed ≥3 times (for an abundant non-secreted structural protein); UMOD (uromodulin) from kidney cells had 15 identifiers in 3 builds, and was observed ≥24,115 times; ALB (albumin) had 18 identifiers in 3 builds, and was observed ≥33,149 times. The times observed could be used as an indicator of relative abundance. UMOD and ALB were two of the most abundant urinary proteins identifiable by gel electrophoresis separation and mass spectrometry of excised protein bands [19]. In Figure 4, urine from a bladder cancer patient B13-026 was tested, and the level of AGR2 was found to be 7.5-fold higher than buffer (note that tumors generally involve only a small part of the urothelium). This suggested that urothelial carcinoma cells could secrete AGR2.

**Figure 5:**
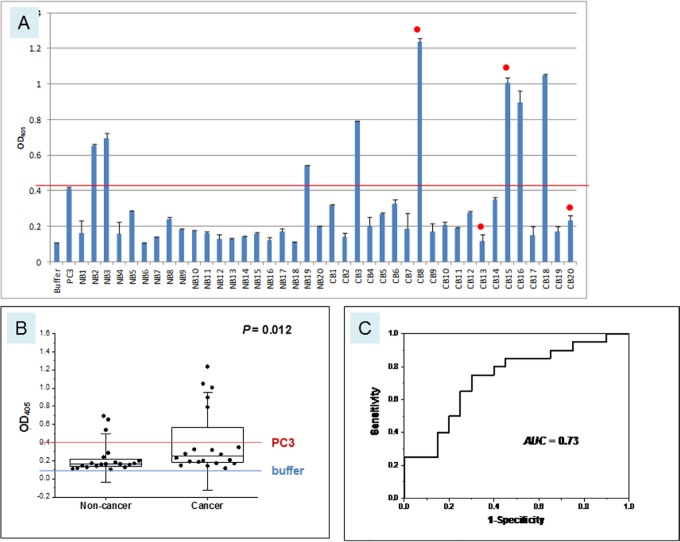
Urinary AGR2 levels in bladder cancer patients **A.** The *x*-axis and *y*-axis are the same as those in Figure 4. Samples labeled NB are non-cancer (n = 20), CB are cancer (n = 20). The red line marks the level of positive control PC3 and the blue line the level of negative buffer control. Red dots indicate cases with recurrence. **B.** The boxplot is shown with the 25 and 75 percentile values and the medians, 1.5xSD values in whiskers. *P* = 0.012. **C.** Shown is the AUC = 0.73.

Urine from a cohort of 20 non-cancer (NB) and 20 cancer (CB) patients (Supplementary Table 2) was tested for AGR2 by ELISA (Figure 5). Using the OD_405_ value for PC3 positive control as a reference, the majority of non-cancer samples were below the reference value, with only 3 (15%) exhibiting higher values. AGR2 in these samples could be due to breakdown of released urothelial cells from normal tissue turnover. In contrast, 5 cancer samples (25%) scored well above the PC3 reference value, a frequency that was expected given the percentage of primary urothelial carcinoma being positive for AGR2 as determined above. These results suggested that AGR2 was secreted by bladder cancer cells and not by normal urothelial cells, despite both cell types expressing AGR2 (*P* = 0.012). The AUC for this cohort analysis was 0.73. Two of the five urine positive cases (40%) suffered recurrence as did two of the urine negative cases (13%).

## DISCUSSION

Unlike the prostate and pancreas where AGR2 is up-regulated in cancer cells compared to normal cells, AGR2 is down-regulated in a majority of bladder cancer cells compared to normal bladder cells. AGR2 expression in normal urothelial cells is comparatively lower than that in prostate cancer cells. Increased AGR2 expression is also found in bladder tumors. Significantly, AGR2 is not secreted by urothelial cells as no large amounts could be detected in the urine of healthy people by sensitive methods such as ELISA and targeted proteomics to pg/ml levels. The proteome of normal urine contains no AGR2. The urothelium also does not secrete AGR2 into blood vessels of the lamina propria as little AGR2 was detected by targeted proteomics in the blood of healthy people [6]. Normal lung epithelium is also positive for AGR2 expression [20]. The lung, like the bladder, apparently does not secrete large amounts of AGR2 into circulation. Otherwise, a substantial level could be measured in blood. High serum AGR2 levels in the ng/ml range reported in the literature were determined by a commercial ELISA based on polyclonal antibodies of suspect specificity [21]. These antibodies detected AGR2 expression in prostate neuroendocrine small cell carcinoma, which was shown by our monoclonal antibodies and DNA arrays to be negative for AGR2 [14]. Targeted proteomics does not have the disadvantage of cross-reactivity in antibodies, and peptide identification is based on unique sequences. AGR2 secretion appears to be a characteristic of cancer cells. Normal bladder non-secretion of AGR2 makes urinary AGR2 measurement a viable test to detect prostate cancer [5]. Different subcellular localization could be due to different AGR2 isoforms, which could result from alternative splicing [22] or differential post-translational modifications. A candidate splice variant (with a deleted *C*-terminal KTEL), however, encodes a much smaller protein missing multiple exons. Alternatively, overexpression of AGR2 in cancer cells could saturate the KDEL receptors in the ER causing the excess protein molecules to be exported. To understand the precise mechanism likely requires purification of the protein from cancer cell cultures for comparison to that purified from normal tissue cells. This cancer specificity would make AGR2 an attractive tumor-associated antigen for therapeutic targeting. In a biodistribution study using radiolabeled anti-AGR2 monoclonal antibody P3A5, an implanted murine Agr2^+^ pancreatic tumor was specifically labeled whereas the bladder, lung (and pancreas) were not (unpublished data of Drs. Laurent Dumartin and Tatjana Crnogorac-Jurcevic, Queen Mary University of London). The anti-human AGR2 P3A5 also recognizes murine Agr2 as the two proteins are highly similar. The result demonstrated absence of cell surface Agr2 in normal cells. With regard to the function of cancer-secreted AGR2, the addition of AGR2-containing media to cultured prostate stromal cells showed that these cells were induced to undergo programmed cell death with cellular blebbing, cell shrinkage, and chromosomal DNA fragmentation. These features were not observed in media containing no AGR2, or when P3A5 was co-added to the media (unpublished data). It is possible that large amounts of free AGR2 in circulation would have a deleterious effect on normal tissue cells.

Bladder tumor AGR2 expression in the TMA cohort was not correlated with patient survival, unlike what has been found in prostate and lung cancer [13, 22]. Both AGR2^+^ and AGR2^−^ tumors were found in cases of short survival, although nearly all of the few long surviving patients with lymph node involvement had AGR2^−^ tumors. Previous limited studies using qRT-PCR, which did not pinpoint the expression location, showed under-expression of AGR2 in bladder tumors, and no correlation with progression or survival [23]. Patients with AGR2 positive lymph nodes did not show lower survival [24]. A trend towards a higher percentage of AGR2^+^ lymph nodes suggests that AGR2-expressing cancer cells could possess a greater potential for local spread. AGR2 expression was up-regulated in a gastric cancer cell subline with high metastatic potential for invasion of lymph nodes [25]. In prostate cancer spread to regional lymph nodes, however, CD10 is more involved than AGR2 [26]. A correlation between higher grade and cancer progression was also reported for CD10^+^ bladder tumors [27, 28], although CD10 expression in our TMA cohort was previously found to correlate with a more favorable outcome [29]. The AGR2^−^ tumor cells of the bladder cancer profiled by our array analysis were positive for CD10. Bladder cancer cells, like prostate cancer cells, could be sorted into different AGR2/CD10 phenotypes for survival correlation [14]. For example, AGR2^high^CD10^low^ would correspond to a different outcome than AGR2^high^CD10^high^. Much larger cohort studies involving over 1,000 cases, which can be difficult to obtain as bladder cancer is not common, as done in prostate cancer regarding cancer expression of CD10 [30] are needed to resolve the divergent conclusions reported in the literature. Interestingly, distinct subcellular expression patterns of CD10 (cell surface *vs*. cytoplasmic) were found to be associated with prostate tumor grade [30]. CD10 cytoplasmic expression could lead to new functions through novel interactions with other proteins such as HSP27 and HSP70 [31]. Differential subcellular localization of protein molecules shown by both AGR2 (cell interior to cell exterior) and CD10 (cell exterior to cell interior) could well be a property of cancer cells. Cell surface or secreted AGR2 likely could also interact with other proteins (e.g., CD10, a cell surface peptidase) to generate new functions. Whether AGR2^+^ tumor cells predominate in the late stages of bladder cancer like prostate cancer [14] remains to be determined. This would require harvesting tumor metastases in advanced diseases as reported by our group for prostate cancer [32].

## MATERIALS AND METHODS

### Patient materials and ethics statement

This study was approved by the Institutional Review Boards of the University of Washington, the University of California, Los Angeles, the University of Bern, Switzerland, and the University of Hawaii. Written informed consent for individual patients was obtained for the use of urine and tissue samples in this research.

### AGR2 immunohistochemistry and bladder tissue microarrays

OCT-frozen benign bladder tissue obtained from surgery at the University of Washington was processed for staining with mouse monoclonal anti-AGR2 clone P1G4 (IgG1) at 1:30 [5]. Negative control was stained with concentration-matched non-immune IgG. AGR2 expression in cancer was evaluated on tissue microarrays (TMA) constructed from primary tumor (each specimen consisted of samplings of tumor center and invasion front) and corresponding lymph node metastases [29]. These tumor samples were obtained from 152 lymph node-positive bladder cancer cases that were treated by cystectomy and pelvic lymphadenectomy at the University of Bern. TMA were immunostained with rabbit polyclonal ab43043 (abcam, Cambridge, MA) at 1:30 following antigen retrieval [13]. Each stained tissue core was examined by a pathologist (LDT), photographed and AGR2 expression was recorded numerically: 1 for weak, 2 for moderate, 3 for strong; plus the percentages of staining as described [13].

### AGR2 expression levels in bladder cells *vs*. prostate cancer cells

Transcriptome datasets of sorted CD9^+^ urothelial cells [10] and CD26^+^ prostate cancer cells [7] were queried for intensity values of AGR2 (i.e., virtual Northern blot analysis). The values of AGR2 and reference housekeeping genes [33] aminolevulinate synthase (ALAS1), β-2-microglobulin (B2M), glyceraldehyde 3-phosphate dehydrogenase (GAPDH), hypoxanthine phosphoribosyl transferase (HPRT1), tubulin (TUBA1) as well as MME (CD10) for comparison were downloaded from the respective transcriptome datasets archived in our public database http://scgap.systemsbiology.net/data/. The values were displayed on a gray scale.

### Bladder cancer AGR2 expression and clinical outcomes

AGR2 expression was correlated with tumor characteristics (stage, extracapsular extension, number and total diameter of metastases) and survival. Briefly, non-parametric two-group and multi-group comparisons were carried out by Mann-Whitney and Kruskal-Wallis tests, and correlations by Spearman Correlation. The Cox proportional hazards model was used for statistical significance of predictors in both a univariate and a multivariate setting. All statistical analyses were performed with StatView Version 5.0 (SAS Institute) or software package R (http://www.r-project.org).

### AGR2 ELISA of voided urine

Urine samples were collected from two young female donors on separate days. The supernatant was desalted and concentrated by Amicon filters (Millipore, Billerica, MA) [5]. An amount of 30-50 ml was concentrated to 600-1,000 μl in 50 mM NH_4_HCO_3_ for measurement of AGR2. The sandwich ELISA was based on generated monoclonal antibodies P1G4 (IgG1) and P3A5 (IgG2a) [5]. Purified P1G4 (1:1,000) was used to coat BD Falcon Flexible plates (BD Biosciences, Mountainview, CA) in phosphate-buffered saline (PBS). The blocking solution contained 1% BSA in PBS, and the urine samples were added at 4° for overnight incubation. Purified P3A5 (1:1,000) was used for detection with horse radish peroxidase (HRP)-conjugated anti-mouse IgG2a and chromogenic substrate 3,3′-diaminobenzidine (DAB). Recombinant AGR2 (rAGR2, GenWay Biotech, San Diego, CA), which was recognized by the antibodies, was employed for calibration. Urine samples from bladder cancer patients were collected during clinical visits (at the University of Hawaii Cancer Center), frozen at −80°, and shipped to UW. The exclusion criteria for male bladder cancer patients included no concurrent prostate cancer diagnosis. The cell-free supernatant of tissue digestion media of the AGR2^+^ prostate cancer xenograft LuCaP 23.12 tumor [14] and the media of cultured AGR2^+^ prostate cancer cell line PC3 [5] were used as positive control. For ELISA data presentation, *OriginPro 2015* (http://www.originlab.com) was used to draw boxplots. The *P*-value was calculated using the non-parametric Wilcoxon-Mann-Whitney test in *OriginPro 2015*. The receiver operating characteristic (ROC) curve was generated to represent the trade-off between the false positive rates and true positive rates for every possible cutoff value. The confidence level was at 95%. The area under the curve (AUC) was calculated with *OriginPro 2015*.

### Urine proteome database query

The human urine proteome datasets archived in *PeptideAtlas* (http://www.peptideatlas.org) were interrogated for mass spectrometry data entries. The *UrinePA* build contained high confidence peptide and protein identifications from five different labs including ours [34]. It contained 2,500 non-redundant proteins cataloged at 1% false discovery rate. A sub-database listed 587 entries of the “Core Urinary Proteome”, which was established from analysis of second morning urine collected over three days from seven young healthy volunteers [35].

## SUPPLEMENTARY TABLES







## Abbreviations

AGR2: anterior gradient 2
ALAS1: aminolevulinate synthase
ALB: albumin
AUC: area under the curve
B2M: β-2-microglobulin
CB: bladder cancer
CD: cluster designation
DAB: 33′-diaminobenzidine
ELISA: enzyme-linked immunosorbent assay
ER: endoplasmic reticulum
GAPDH: glyceraldehyde 3-phosphate dehydrogenase
HPRT1: hypoxanthine phosphoribosyl transferase
HRP: horse radish peroxidase
MME: membrane metallo-endopeptidase
NB: normal bladder
OCT: optimal cutting temperature compound
pT: pT4 pN2 bladder cancer stages
PDI: protein disulfide isomerase
rAGR2: recombinant AGR2
ROC: receiver operating characteristic
SD: standard deviation
TMA: tissue microarrays
TUBA1: tubulin
UMOD: uromodulin
UPK3A: uroplakin
