# Supplementary material for: Bladder cancer cells secrete while normal bladder cells express but do not secrete AGR2
Source: Oncotarget. 2016 Feb 15;7(13):15747–56. doi: 10.18632/oncotarget.7400 (PMC4941274; doi:10.18632/oncotarget.7400)
Supplement: Supplementary file 1 [file oncotarget-07-15747-s001.pdf]

SUPPLEMENTARY TABLES

Supplementary Table 1: Patient cohort used for the TMA. Intensity of staining is indicated by color hues. Blank signifies no staining, and gray signifies no data.

See Supplementary File S1

Supplementary Table 2: Pathology of bladder cancer patients in urine AGR2 analysis. Where available, the stage, grade, tumor size (in cc) of the urothelial carcinoma are given. Ta = noninvasive papillary; Tis = noninvasive flat carcinoma in situ. The CB cases highlighted are known to recur. In race, W = Caucasian, H = Hispanic, B = African American, A = Asian. Follow-up is in months. TCC = transitional cell carcinoma. PUNLMP = papillary urothelial neoplasm of low malignant potential.

See Supplementary File S2
