# Supplementary material for: Bladder cancer cells secrete while normal bladder cells express but do not secrete AGR2
Source: Oncotarget. 2016 Feb 15;7(13):15747–56. doi: 10.18632/oncotarget.7400 (PMC4941274; doi:10.18632/oncotarget.7400)
Supplement: Supplementary file 2 [file oncotarget-07-15747-s002.pdf]

patient	AGR2<br>tumor center	tumor invasion front	lymph node mets I	lymph node mets II	overall survival yrs	gender	pT	pN	capsule perforation
B86-01					1.9	m	3	2	1
B86-02					1.6	m	3	2	1
B86-03					0.8	m	4	2	1
B87-01					1.3	m	4	2	1
B88-01					4.8	m	2	2	0
B89-01					0.4	f	3	2	1
B89-02					4	m	3	2	1
B90-01					18.9	m	3	2	1
B90-02					3	m	4	2	1
B90-03					18.6	m	4	2	0
B90-04					0.4	m	4	2	1
B90-05					3	f	3	2	1
B91-01					0.4	m	4	2	0
B91-02					15.8	m	3	2	0
B91-03					0.2	m	3	1	0
B91-04					5.3	f	3	2	0
B91-05					14.6	m	4	2	0
B92-01					7.8	m	3	2	0
B92-02					8.3	m	3	2	1
B92-03					1.5	m	4	2	1
B92-04					14.1	m	1	2	0
B92-05					2.5	m	4	1	1
B93-01					0.2	m	4	2	1
B93-02					0.2	m	3	2	1
B93-03					1.1	m	3	2	0
B93-04					5.1	m	3	2	1
B93-05					7.3	m	2	3	1
B93-06					0.6	m	3	2	1
B94-01					14.5	f	4	2	0
B94-02					1.9	m	3	2	1
B94-03					14.6	f	3	2	0
B94-04					1	f	4	2	1
B94-05					0.1	m	3	2	1
B94-06					1.1	m	4	2	1
B95-01					0.7	m	4	2	1
B95-02					4.2	m	3	2	1
B95-03					4.5	m	3	3	1
B95-04					5.2	f	3	2	1
B95-05					3.1	m	3	2	0
B95-06					4.1	m	3	2	0
B96-01					6.2	m	4	2	1
B96-02					1.2	f	4	2	1
B96-03					0.4	m	4	2	1
B96-04					9	f	3	2	0
B96-05					1.6	m	3	3	1
B96-06					1.9	f	3	2	1
B96-07					2.1	m	4	2	1
B96-08					0.4	m	3	2	1
B96-09					0.7	m	3	2	0

B96-10					8.8	m	2	2	0
B97-01					1.9	m	4	2	1
B97-02					1.3	m	3	2	1
B97-03					1	m	4	3	1
B97-04					0.7	m	3	2	0
B97-05					0.6	m	3	1	0
B98-01					0.8	m	4	3	0
B97-06					0.1	m	3	2	1
B98-02					0.1	m	3	2	1
B98-03					10.9	m	3	2	0
B98-04					0.5	m	3	3	1
B98-05					0.6	m	2	1	0
B98-06					10.9	m	3	1	0
B98-07					1.1	m	4	1	1
B98-08					2.1	m	3	2	1
B98-09					1.2	f	3	1	1
B98-10					1	m	3	1	0
B98-11					2.8	f	3	2	1
B99-01					10	m	2	2	0
B99-02					5.8	f	3	2	1
B99-03					1.8	m	3	2	1
B99-04					2.4	m	3	1	0
B99-05					0.2	m	3	2	1
B99-06					1.6	m	3	2	1
B00-01					0.2	m	3	2	1
B00-02					0.2	m	4	2	0
B00-03					4.2	f	2	2	0
B00-04					6.8	m	3	2	1
B00-05					0	m	4	2	1
B00-06					3.2	m	3	2	0
B00-07					7.6	m	3	2	0
B00-08					0.3	m	4	2	1
B00-09					0.9	m	3	2	1
B01-01					7.5	f	2	2	0
B01-02					0.7	m	4	2	0
B01-03					8	m	3	2	0
B01-04					8	m	2	1	0
B01-05					n/a	m	3	2	0
B01-06					0.7	m	4	2	1
B01-07					0.9	m	4	3	1
B01-08					2.6	m	3	2	0
B01-09					7.1	m	1	1	1
B01-10					7.1	m	4	2	1
B01-11					7	m	2	1	0
B02-01					7.1	m	1	2	0
B02-02					1.4	m	3	2	0
B02-03					0.3	m	3	2	1
B02-04					1.1	m	2	1	0
B02-05					6	f	3	2	0
B02-06					1.3	m	3	2	0
B02-07					0.1	m	4	2	1
B02-08					1.5	m	3	1	0

B03-01					3.7	m	2	2	0
B03-02					0.8	m	3	2	0
B03-03					6	m	2	1	0
B03-04					4.1	m	2	1	0
B03-05					1	m	4	2	0
B03-06					2.1	m	3	2	0
B03-07					0	m	3	2	1
B03-08					4.6	m	3	2	0
B03-09					1.5	m	1	1	0
B03-10					1.3	m	3	2	1
B03-11					5	m	3	1	0
B04-01					2.4	m	3	1	0
B04-02					1.3	f	3	2	1
B04-03					2	f	4	2	1
B04-04					4.8	f	4	2	1
B04-05					0.9	f	3	2	0
B04-06					2.8	m	2	2	1
B04-07					0.2	m	4	2	0
B04-08					4.4	m	3	1	0
B04-09					0.7	f	3	3	1
B04-10					0.1	m	3	2	0
B04-11					1.7	m	4	3	1
B04-12					2	m	3	2	1
B04-13					4	m	3	2	1
B04-14					4.1	f	3	2	0
B05-01					4.1	f	3	2	0
B05-02					0.5	f	3	2	0
B05-03					2	m	4	1	0
B05-04					1.8	m	3	2	1
B05-05					1.3	m	3	2	1
B05-06					2.9	f	3	1	0
B06-01					1.2	m	2	3	1
B06-02					0.4	m	3	2	0
B06-03					2.4	f	3	1	0
B06-04					2.6	m	2	1	0
B06-05					2.5	m	3	3	1
B06-06					2.8	m	3	1	1
B07-01					0.6	m	3	3	1
B07-02					2	m	3	1	0
B07-03					2	m	4	1	0
B07-04					1.9	f	3	1	1
B07-05					1.6	f	3	3	1
B07-06					1.8	m	2	3	0
B07-07					1.6	m	3	1	0
B07-08					1	m	3	3	1
B07-09					1.3	m	3	3	1
B07-10					0.9	m	3	3	1
B07-11					0.3	m	4	3	1
B08-01					1	m	3	2	0
B08-02					1	f	3	2	1
B08-03					1	m	3	1	0

strong 3+
moderate 2+
weak/focal 1+
faint 0+
negative
no data

survival >10 y
survival <1 y
