# Supplementary figures and images for: Bladder cancer cells secrete while normal bladder cells express but do not secrete AGR2
Source: Oncotarget. 2016 Feb 15;7(13):15747–56. doi: 10.18632/oncotarget.7400 (PMC4941274; doi:10.18632/oncotarget.7400)

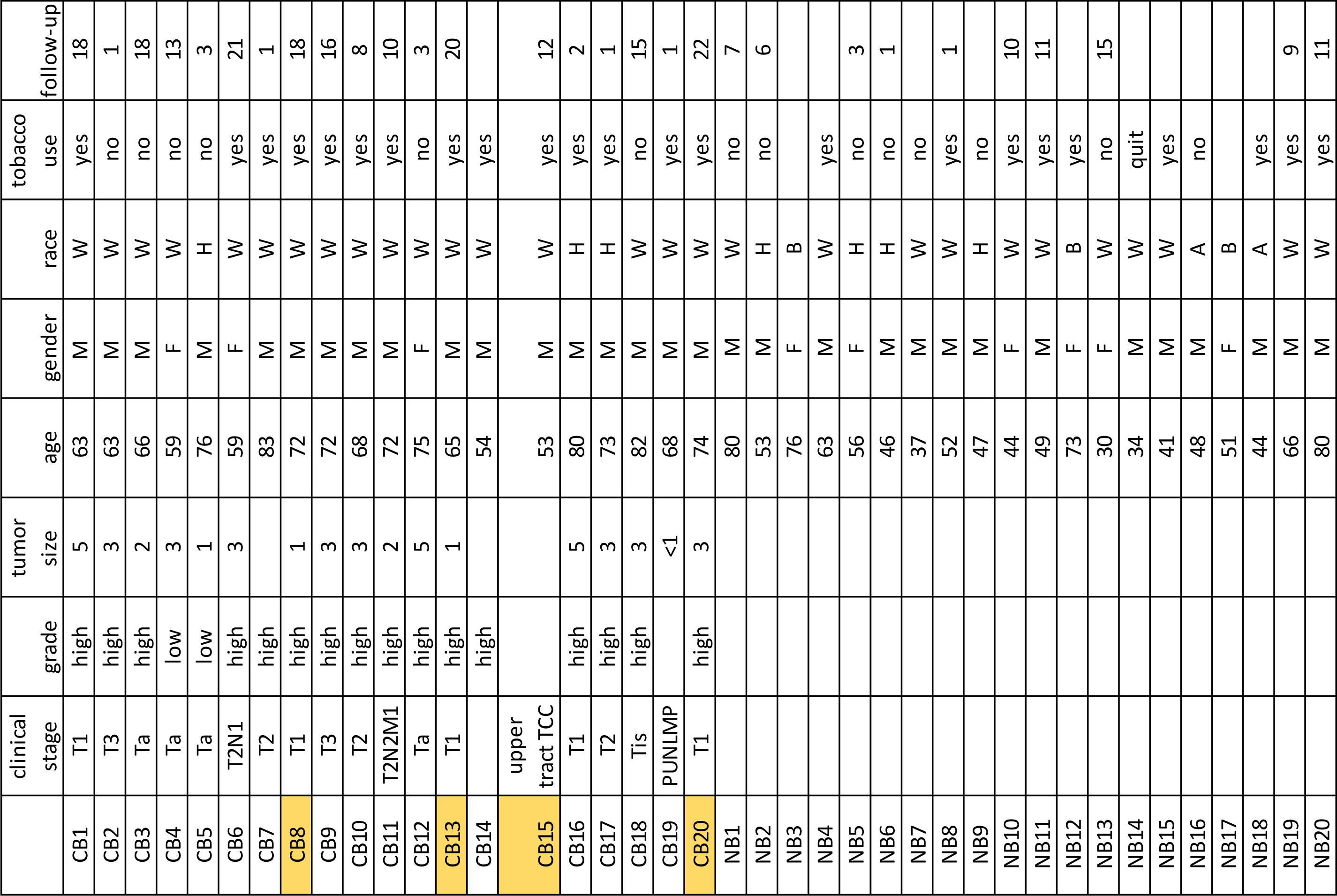

Supplement: Supplementary file 3 [file oncotarget-07-15747-s003.tif]
